# Evaluating Potential Deployment Strategies for Oral Delivery of Vaccines for Cervids

**DOI:** 10.1155/tbed/5900650

**Published:** 2026-05-28

**Authors:** Amanda S. Zimmerling, Scott Napper

**Affiliations:** ^1^ One Health Medical Technologies, Saskatoon, Saskatchewan, Canada; ^2^ Vaccine and Infectious Disease Organization (VIDO), University of Saskatchewan, Saskatoon, Saskatchewan, Canada, usask.ca

**Keywords:** bait preference, cervid, chronic wasting disease, nontarget species, oral delivery system, trail camera, vaccine

## Abstract

While vaccination is the most efficacious strategy for landscape‐scale disease management, its success is predicated on overcoming concurrent hurdles in both laboratory‐scale vaccine development and the logistical and behavioral complexities of field delivery to targeted wildlife populations, such as cervids. As laboratory effectiveness and successful field deployment of vaccines are interdependent, these efforts should be performed concurrently, with findings in one influencing progress in the other. A proactive approach for development of effective deployment strategies also minimizes any logistical bottlenecks that could occur following vaccine regulatory approval. This multiregional field study utilized a rotational crossover design across 19 sites in Alberta and Saskatchewan (May–August 2025) to quantify the behavioral efficacy of two candidate vaccine delivery matrices (thin film and liquid‐filled packets [boba]) in combination with a variety of common cervid attractants. Remote camera monitoring was employed to assess target cervid (deer, elk, and moose) foraging preference, nontarget species uptake, and individual repeat visitation frequency. The thin‐film matrix, presented within peas as food attractant, was found to be the optimal bait‐delivery matrix maximizing target uptake. This analysis simultaneously quantified two critical operational hurdles (1) high nontarget consumption and (2) repeat consumption, or large‐dose consumption, by individual cervids. These behavioral constraints invalidate a generic scatter‐bait approach and underscore a challenge in delivery engineering. These findings provide the quantitative parameters required to support the development of a “smart” oral delivery system that incorporates remote species recognition and portion control to achieve cost‐effective, single‐dose delivery in wild populations.

## 1. Introduction

Over the past decade, interest and investment in oral vaccination strategies have sharply increased [1], largely driven by the operational success of wildlife rabies vaccination programs [2, 3]. This method, which involves dispersing liquid vaccine packages coated in an attractant, offers a scalable and economic alternative to capture‐and‐inject delivery methods. While the success of the oral vaccination programs for rabies highlights the potential for control of wildlife diseases through oral vaccination, there are species‐specific nuances to the required vaccine delivery system. Specifically, the packaging and baiting approaches of the oral rabies vaccines, which are optimized for carnivores and scavengers like raccoons and coyotes, are incompatible with the foraging habits of other species, particularly grazers such as cervids. This incompatibility necessitates substantial research into developing species‐specific oral delivery systems.

Oral vaccine deployment, while the most favorable approach to wildlife vaccination, introduces its own unique and complex set of challenges rooted in behavioral ecology that must be resolved before deployment [4, 5]. Two critical interrelated hurdles are achieving targeted dose‐controlled uptake by the desired species and ensuring the avoidance of nontarget exposure. While laboratory research primarily prioritizes the development of efficacious vaccines, the capacity to successfully deliver these vaccines to target species while simultaneously minimizing waste from both repeat individual and nontarget uptake has received comparatively less scientific consideration. This likely reflects both a sparsity of examples of vaccines, which have been translated into commercial products as well as a tendency to underestimate the complexities associated with effective delivery systems. Establishment of effective vaccines and vaccine delivery systems is not isolated, independent activities; decisions in each activity will have consequences for the other. Accordingly, these efforts are best performed in parallel, with progress in each realm guiding and informing the other. Establishing an effective delivery system is also essential for increasing regulatory support and governmental buy‐in for wildlife vaccination programs and for reducing overall project costs through optimized delivery methods.

This challenge was the stimulus for the current project. This work was carried out in conjunction with ongoing laboratory research into the development of an oral vaccine for chronic wasting disease (CWD), a fatal prion disease rapidly spreading across cervid populations [6, 7]. CWD threatens the ecological stability of species within the Cervidae family, including white‐tailed deer (*Odocoileus virginianus*), mule deer (*Odocoileus hemionus*), elk (*Cervus canadensis*), and moose (*Alces alces*) [8]. Pioneering work in the research community has led to the promising development of oral CWD vaccines in various stages of trials [5, 9]. Given that cervid feeding habits are incompatible with currently deployed wildlife vaccination strategies, this deployment research is necessary for eventual successful field use.

Utilizing a multiregional field study across diverse landscapes in Alberta and Saskatchewan, the Canadian provinces with the highest rates of endemic CWD, two oral delivery methods, encapsulated liquid (boba) or dry film strips, both as biologically inactive placebos, were combined with various common cervid baits/livestock feeds. Remote camera monitoring was employed at each site to quantify behaviors around bait consumption.

Specific objectives of the study were as follows:

1. Quantify the relative feeding preferences of the target cervids across the various delivery matrices to identify optimal formulations for maximum target uptake.
2. Quantify nontarget species uptake to assess potential waste.
3. Determine the frequency of repeat uptake by individual cervids to model effective dosage and target species‐related vaccine waste.

By providing quantitative data on the logistical efficacy and ecological safety of oral bait systems, the findings aim to directly inform vaccine development, refine field deployment protocols for cervid oral vaccines, and establish a generalizable framework for scaling the requirements for a potential “smart” oral vaccine delivery system outfitted with means to reduce nontarget and repeat uptake.

## 2. Study Area

**Figure 1:**
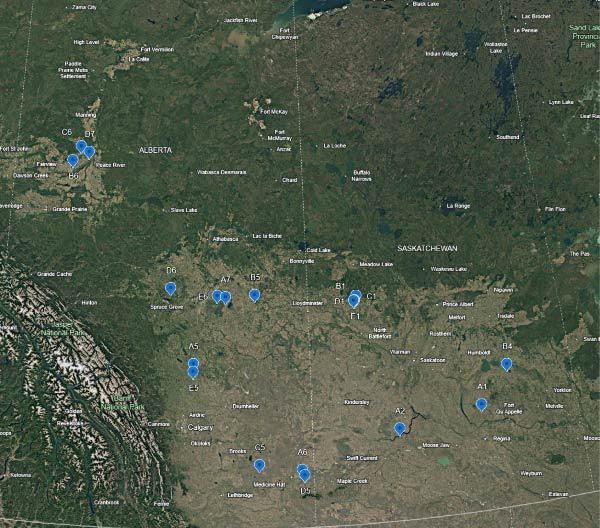
Map locations for 19 trial site locations with remote camera monitoring spanning Saskatchewan and Alberta.

Study areas were primarily situated within the prairie and boreal transition ecozones of central and southern Alberta and Saskatchewan (Figure 1). While CWD has been detected in five Canadian provinces, Saskatchewan and Alberta were prioritized, as the vast majority of these cases, in particular within wild animals, occur within these two provinces [10]. This region is characterized by flat to gently rolling topography, with the study sites representing a mosaic of mixed‐grass prairie, cultivated agricultural lands, and aspen parkland. All sites were established on private land with explicit permission from the respective landowners; however, several locations were associated with conservation areas that allow public access.

Encompassing the late spring and summer seasons in the Canadian prairies (from May 5th to August 31st), study sites incurred mild to warm temperatures. While overall precipitation can be considered average for the time frame of these experiments, central and northern Alberta and Saskatchewan both received significantly less than average rainfall, while the southern reaches of the province received above‐average precipitation [11, 12]. This study period coincided with an active wildfire season in Western Canada, resulting in the second worst wildfire season in Canada’s history [13]. Multiple test sites were periodically subjected to reduced air quality and atmospheric smoke, particularly during June and early July. Although no sites experienced direct fire impact, the ambient environmental stressor of regional wildfires is known to impact wildlife behavior.

The primary target species for the bait systems were white‐tailed deer, mule deer, elk, and moose. Expected nontarget species included black bears (*Ursus americanus*), coyotes (*Canis latrans*), foxes (*Vulpes vulpes*), and various small rodents and birds.

## 3. Methods

### 3.1. Bait and Site Selection

**Figure 2:**
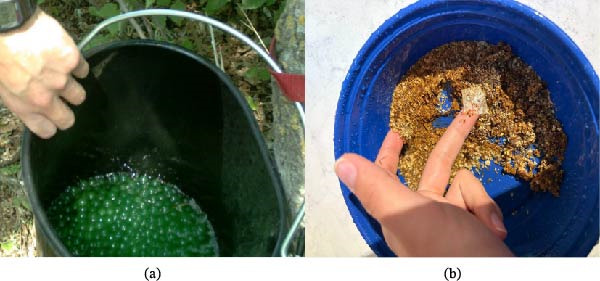
Encapsulated liquid placebo (boba) (a) and inert dry film mixed with feed (b).

The experimental design utilized a rotational approach. Treatment combinations were systemically rotated across established sampling units over defined 2‐week intervals. Treatment groups consisted of two primary placebo vaccine delivery matrices combined with common cervid attractants and feeds. The first vaccine format investigated was an encapsulated liquid placebo (Green Apple Popping Boba, AB Distribution, Calgary, AB, Canada), while the second was an inert dry film (Jurata, Texas, USA) [14, 15] (Figure 2).

These matrices were combined with compatible base feeds including peas (Yellow Feed Peas, Early’s Farm and Garden Centre, Saskatoon, SK, Canada), alfalfa cubes (Alfalfa Cubes, Early’s Farm and Garden Centre, Saskatoon, SK, Canada), and mineral licks (Redmond Rock and Mineral Lick and Cobalt Iodized Salt Lick, Early’s Farm and Garden Centre, Saskatoon, SK, Canada), which were selected based on known palatability to cervids and common usage by outfitters and livestock producers. This led to a total of 11 treatments, including controls, as outlined in Table 1.

**Table 1:** Description of all treatments consisting of various placebo and bait mixes.

Treatment	Description
Control	Full site setup with no bait or vaccine placebo
Liquid placebo	Liquid placebo in feed bucket
Dry film	Dry film in feed bucket
Mineral lick	Mineral lick placed above feed bucket
Peas	Dried livestock feed peas in feed bucket
Alfalfa	Alfalfa cubes in feed bucket
Liquid placebo + mineral lick	Mineral lick placed above feed bucket containing liquid placebo
Liquid placebo + peas	Peas and liquid placebo mixed in feed bucket
Dry film + mineral lick	Mineral lick placed above feed bucket containing dry film
Dry film + peas	Peas and dry film mixed in feed bucket
Dry film + alfalfa	Alfalfa and dry film mixed in feed bucket

Each site was classified into one of five treatment rotations (A–E) with the goal of achieving a 16‐week experimental time frame per site. Each complete rotation cycle, lasting 8 weeks, consisted of a control, bait‐only, placebo‐only, and placebo plus bait period, with all treatment periods set to occur for 2 weeks before repeating the cycle (see Supporting Information: Supporting Table, Appendix A—A5 Site Details).

Due to resource constraints, the site location was primarily determined through directed convenience sampling. Participation was solicited from landowners, outfitters, and wildlife biologists/conservation organizations with expert knowledge of cervid behavior and access to locations of high‐density cervid populations across Alberta and Saskatchewan. Targeted outreach was utilized to attempt to garner as wide a geographical range across provinces as possible. While 25 sites were originally secured, reliance on volunteer contributions and difficulties in scheduling resulted in 19 sites being included in the analysis for this study.

### 3.2. Site Setup

Each bait station was designed to maximize the potential detection of target and nontarget species while allowing for quantification of both site visits and consumption events. Collaborators selected suitable locations on their property known to exhibit high cervid activity (e.g., adjacent to game trails or bedding sites). Livestock feed buckets were placed on the ground or slightly elevated and secured in that location to allow for easy cervid feeding.

Each site consisted of the deployment of two motion‐activated cameras of various makes and models (often volunteer‐supplied) to ensure redundant coverage and comprehensive data collection. The first camera was mounted ~4–5 m from the feed vessel aiming perpendicular to the expected animal movement path at an approximate deer shoulder height to achieve a wide field of view, capturing both animals at the feed station and those that just pass by. The second camera was mounted ~1–2 m from the feed bucket and was angled to provide a view into the feed bucket itself. This deployment was used to monitor consumption events and feed volume. A full site setup plan can be found in Supporting Information: Appendix B.

### 3.3. Data Collection

All remote cameras were configured to operate in image mode, capturing one photo per trigger with a 5–20 s interval between consecutive triggers. Data for each camera were collected on secure digital (SD) cards before being uploaded to a shared Google Drive for analysis. All captured images were reviewed for three primary behavioral metrics and recorded on a master spreadsheet.

1. Species identification: Identification of all species visiting the bait station and categorization into target vs. nontarget.
2. Consumption metrics: Quantification of the number of individuals of the target and nontarget species interacting with the bait.
3. Repeat uptake: When possible, the same individual cervids were identified (generally by antler patterns nearing the end of the test cycle).

Deployment and servicing data were recorded and submitted by each volunteer to ensure functionality at each treatment changeover (Supporting Information: Appendix A–C, Appendix 1).

### 3.4. Statistical Analysis

JASP Statistical Analysis Software was for all statistical analysis. Data were aggregated for each 2‐week time period and normalized per day of treatment to account for differences in days of each treatment available, resulting in one data point per treatment‐period‐site combination. Generalized linear mixed model (GLMM) approaches were utilized to accommodate the normalized count data and the repeated measure design of the rotation study. Pearson goodness‐of‐fit tests were used to confirm that the models were sound. Following the GLMM, Tukey’s post hoc tests were utilized to determine specific pairwise differences between treatments.

## 4. Results

### 4.1. Overall Visitation and Data Summary

Studies were highly successful, with 119 treatment periods being analyzed over 18 different sites encompassing 1320 total visitation events. White‐tailed deer were the most frequently observed species (53% of all visits and 77% of all target visits). A breakdown of the various total image captures is summarized in Table 2.

**Table 2:** Summary of total image captures including total counts, total feed counts, percentage of feeding within species, and percentage of feeding out of total consumption.

Species	White‐tailed deer	Mule deer	Moose	Elk	Black bear	Misc. nontarget
Total count	696	84	79	48	100	315
Feed count	139	3	25	15	62	158
Percentage feeding out of species imaged	20%	4%	32%	31%	62%	50%
Percentage feeding out of total consumption	35%	1%	6%	4%	15%	39%

Other than the species listed above, miscellaneous species identified included coyotes, squirrels (Sciuridae family), including flying squirrels (*Glaucomys sabrinus*), weasels (Mustelidae family), skunks (*Mephitis mephitis*), racoons (*Procyon lotor*), snowshoe hares (*Lepus americanus*), and a variety of birds. Birds and squirrels were the most common nontarget animals imaged. While target species were imaged more frequently than nontarget species, nontarget species accounted for 54% of all animals imaged feeding.

### 4.2. Target Preference and Treatment Efficacy

**Figure 3:**
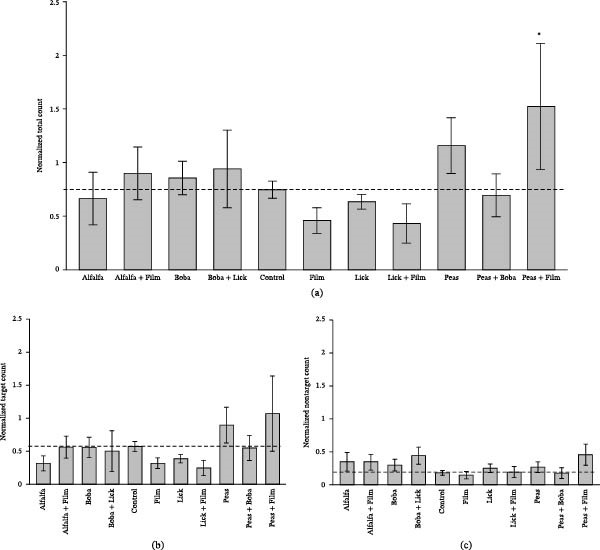
Comparison of overall site visits normalized per day of treatment availability (a), and breakdown by target (b) and nontarget (c) species. Significance is indicated by  ^∗^ *p* < 0.05, and the dashed line indicates the normalized control mean.

Interestingly, overall visits by both target and nontarget species did not increase significantly based on having feed available (*p* = 0.077) compared to the control, with only the feed condition of peas + film indicating a significant increase in overall animal activity (*p* = 0.027) (Figure 3).

**Figure 4:**
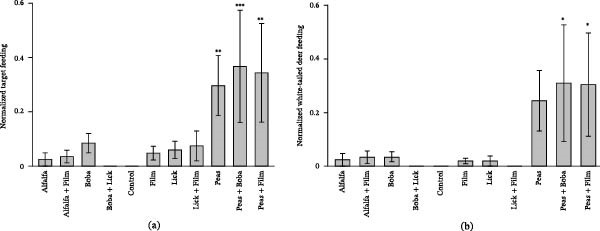
Comparison of normalized target feeding by treatment overall (a), and breakdown for white‐tailed deer (b). Significance is indicated by  ^∗^ *p* < 0.05,  ^∗∗^ *p* < 0.025, and  ^∗∗∗^ *p* < 0.01.

It was found that there was a significant increase in target feeding behavior for all treatments containing peas: peas *p* = 0.017, peas+boba *p* = 0.009, peas + film *p* = 0.019 (Table 3). No other significant differences were identified between the various bait and feed mixtures by the target species, indicating that peas are the favorable bait choice for the target species out of the options tested (Figure 4).

**Table 3:** Tukey’s post hoc comparison of treatments including peas.

Treatment comparison	*p*
Peas−peas + boba	0.998
Peas−peas + film	1.000
Peas+boba−peas + film	1.000

Breaking this data down further by species, white‐tailed deer also demonstrated significant preference for pea‐based treatments, with *p* = 0.029 and *p* = 0.040 for peas + boba and peas + film, respectively (peas *p* = 0.052) (Figure 4). No other significant increases were found within the feeding habits of other species monitored, including nontarget species.

### 4.3. Effect of Repeated Measures

**Figure 5:**
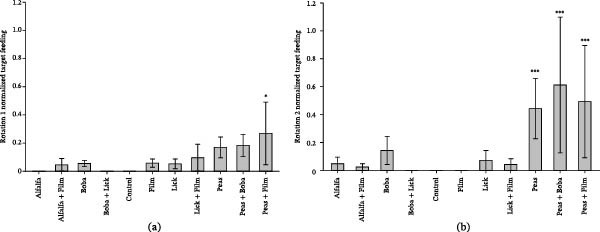
Comparison of normalized target feeding behavior between Rotation 1 (a) and Rotation 2 (b). Significance is indicated by  ^∗^ *p* < 0.05 and  ^∗∗∗^ *p* < 0.01.

As these different treatments were rotated through comparison between Rotation 1 (the first occurrence of a specific treatment) and Rotation 2 (the second occurrence of a specific treatment), the difference between occurrences of treatments was analyzed. In the first rotation, only peas + film demonstrated significance (*p* = 0.041); however, during Rotation 2 all pea‐containing treatments demonstrated a significant increase against the control (Figure 5).

### 4.4. Effect of Placebo Vaccine Addition

With the identification of peas as the optimal bait, analysis was also carried out to determine whether mixing the peas with various vaccine mimics resulted in a change in animals’ feeding preference for peas. This was found to not be the case, with post hoc comparisons demonstrating no significant difference and Figures 4 and 5 demonstrating that target feeding generally slightly increased with the addition of the vaccine mimics to the base pea treatment.

### 4.5. Individual Repeat Visitation

Analysis of images captured late in the experimental period allowed for the determination of repeat visitors by antler identification. Thirty‐six time periods were analyzed for repeat individuals, and in 21 periods, repetitive animals were identified (58%). In eight other cases, repeat visitation is suspected (doe‐fawn visitation pairs); however, it could not be confirmed through visual analysis.

## 5. Discussion

The management and control of infectious diseases in wide‐ranging wildlife populations represents one of the most significant and complex challenges in modern conservation and public health [16, 17]. Traditional wildlife disease intervention strategies, including means of population reduction (hunting, culling, and translocation) [18–20] or traditional injection‐based vaccine deployments, are logistically difficult, financially prohibitive, and frequently precipitate ethical concerns and strong political and public opposition [21, 22]. Furthermore, the increasing prevalence and understanding of zoonotic spillover risk underscore the necessity for proactive disease mitigation in wild populations [23, 24]. Notorious examples, such as COVID‐19 [25] and the current global concern over highly pathogenic avian influenza (H5N1) jumping from wild birds to domestic poultry and mammals, emphasize this interconnected vulnerability [26]. Consequently, the development of vaccines that can be administered noninvasively and autonomously in the field is a critical research and management priority.

While this project was designed and executed concurrently with CWD vaccine development efforts, aiming to proactively bridge the critical gap between laboratory efficacy and field deployment success, the experimental architecture and findings are widely applicable to a variety of infectious diseases and oral vaccines under development for cervids, including bovine tuberculosis (*Mycobacterium bovis*), brucellosis (*Brucella* spp.) or anthrax (*Bacillus anthracis*) [27, 28, 29]. In some cases, like that of epizootic hemorrhagic disease virus (EHDV), an injectable vaccine already exists for cervids and is used extensively in the farmed cervid industry but has not been translated to vaccinate wild populations [30]. Areas in Europe that vaccinated up to 80% of farmed disease‐susceptible species demonstrated a resulting disappearance of clinical disease [30]. While these vaccine programs are highly beneficial in negating economic losses due to EHDV, in areas with a high density of wild cervids, this approach is likely to have a minimal impact on rates of disease in wild populations and a limited impact on the spread of the disease to novel areas. Current oral vaccine experimental programs often rely on manual grid‐baiting strategies, utilizing multiday conditioning, followed by vaccine‐inclusive bait distribution [29]. While these methods have demonstrated localized success, they remain inherently limited by high labor costs, restricted geographic scalability, and significant interspecific competition for bait. Even under highly controlled conditions, nontarget consumption and dose monopolization by individual animals remain critical points of failure. This research addresses these deficiencies by providing the empirical behavioral parameters required to transition from manual, high‐intervention baiting to autonomous, precision‐engineered delivery systems.

The scope of this experimental design may be extended beyond cervids to include other free‐ranging North American grazers, such as bison (*Bison bison*) and pronghorn (*Antilocapra americana*), who are also being monitored for various diseases such as bovine tuberculosis and EHDV among others [31, 32]. By quantifying foraging preferences, repeat visitation frequencies, and nontarget interference, this design establishes a high‐fidelity model applicable to any ruminant population where oral delivery is required. Ultimately, these findings serve as a robust technical foundation for future delivery strategies, ensuring that emerging vaccine candidates can be deployed via “smart” systems that overcome the inherent behavioral and logistical barriers of landscape‐scale wildlife health management. Primary findings from this study demonstrate that target cervid consumption of the vaccine placebo is significantly influenced by the bait‐delivery matrix. Among the primary feeds tested (mineral licks, alfalfa, and peas), peas were found to be the preferred bait. Peas could be mixed with either the dry film or liquid vaccine delivery matrix without impacting consumption by the target species compared to the bait‐only control. However, this study only focused on these primary feeds, selected based on cost and professional outfitter recommendations. Common alternative baits, including apples, corn, and various commercial and homemade deer attractant mixes, were not tested. Future studies, building off the work completed here, may wish to test additional possible bait‐delivery matrices to further identify feed preference and the potential for bait selection to decrease nontarget uptake.

This study quantified two major obstacles to simple field deployment of wildlife oral vaccines: waste due to nontarget species uptake and repetitive dosing of individual animals. While the biological risk associated with nontarget uptake has been determined to be minimal [5], the scale of nontarget consumption identified (greater than 50% of feeding animals) suggests potential vaccine waste and corresponding financial cost may render current field deployment strategies nonviable. Black bears were identified as a common nontarget species (15% of feeding animals) and often consumed over 1/3 of the total feed within one visit. Squirrels were also found to have a major impact. At one site, squirrels were the only identified visitor over a 2‐week period, yet the full portion of feed was consumed. While physical barriers like fencing are often proposed to exclude nontarget animals, they create a difficult design conflict between restricting small, agile species and maintaining access for large wildlife. Persistent nontargets such as squirrels are notoriously difficult to keep out, while the target cervids are frequently deterred by the presence of new, complex structures. These limitations indicate that passive fencing likely cannot provide the species‐specific precision required for effective vaccine delivery.

While these experiments were conducted during the spring and summer, data on cervid and bear behavior, indicate that winter would likely be the optimal period for vaccine deployment [33–35]. Bear activity will be significantly decreased, and reduced natural food supplies will be expected to promote cervid visitation to vaccine deployment stations. However, shifting the deployment period to winter is not expected to significantly mitigate waste from other nontarget species. Further, over 58% of sites analyzed for individual feeding repetition demonstrate that target animals will return to the same feeder repetitively, and that number is expected to be an underestimate due to the lack of ability to identify individual animals via human inspection of trail camera images. This repetition would also result in increased financial cost, impacting the viability of field deployment. Further, dose control will need to be considered. While some animals consumed only small portions, other animals would remain feeding at the site for hours at a time, consuming well over what would be required for a successful vaccine dose.

Limitations of this study include a relatively small geographic area and limited bait selections. While regions of Alberta and Saskatchewan were well represented in this study, there was a clear difference between populations of white‐tailed deer and mule deer surveyed, likely due to bait station placement, which should be further investigated to get a better survey of cervid populations [36]. Combining site selection with species demographic and density data, instead of reliance on volunteer availability and locations, could further increase the target uptake and correspondingly decrease nontarget consumption. This would require a high degree of buy‐in from several stakeholders but would optimize deployment location selection. Further, infectious diseases in cervids are not just a Canadian or North American problem. For example, CWD has been detected in free‐ranging cervids in 36 US states, with other cases detected in Scandinavian countries [6, 37]. As any vaccination strategy geared towards containing the spread of infectious diseases in wildlife will need to account for the entirety of the disease’s range, the behavioral characteristics of target species across the entire range should be studied. Unique characteristics across various priority regions for vaccine deployment may benefit from different strategies of food formulation and/or patterns of seasonal distribution; there may not be a single best approach. Notably, the oral vaccines used for the control of rabies in wildlife populations in North America employ different food attractant systems as well as unique biological vectors [38]. Different baits may have a different target vs. nontarget uptake profile, allowing bait selection to exclude a degree of nontarget uptake. However, it is unlikely that bait selection would be capable of removing a large value of nontarget feeding due to the selective feeding habits of cervids in comparison to more opportunistic scavengers. Further, as only 19 sites were able to be analyzed over five different treatment classifications, this study has relatively small statistical power. Narrowing the number of treatments studied while increasing the number of sites would improve data reliability but would require further financial investment and/or collaboration.

## 6. Research Implications

The development of effective oral vaccines against high‐consequence wildlife diseases is progressing in the laboratory. However, a fundamental operational disconnect exists between achieving high vaccine efficacy in controlled laboratory settings and ensuring successful, cost‐effective deployment in the field. This multiregional study was designed to proactively bridge this operational gap by quantifying the foundational behavioral parameters that directly influence field deployment, logistics, and success. The data collected offer metrics that will help inform elements including bait selection, deployment timing, and the necessary design requirements for vaccine delivery systems to minimize waste and the corresponding cost. Further, this data may aid in preempting various regulatory hurdles through understanding nontarget consumption.

Overall, this quantitative behavioral data justifies further deployment engineering. To achieve effective, cost‐efficient, and ecologically safe deployment, efforts must include dose control and species exclusion. The data derived from this multiregional field trial strongly support the development and validation of a “smart” oral vaccine delivery system. This system must incorporate (1) remote species recognition (e.g., AI‐powered cameras) to dispense the dose only upon detecting a target cervid, (2) mechanical portion control (e.g., a timed‐release or physical mechanism), and (3) individual animal recognition to prevent over‐consumption by frequent visitors, thereby maximizing single‐dose delivery across the population. Further benefits of utilizing a “smart” delivery system with these features include the ability to provide vaccines on a vaccine schedule, such as providing a booster dose to an individual animal after a set period. This provides greater flexibility in vaccine efficacy requirements to reach the deployment stage.

Successfully linking these ecological observations with advanced engineering is the most promising path to ensure feasible, cost‐effective, and ecologically safe infectious disease control in wild cervid populations.

## Funding

This study was supported by the Alberta Conservation Association.

## Disclosure

One Health Medical Technologies may see a long‐term benefit from the development and commercialization of “smart” wildlife vaccine stations.

## Ethics Statement

This work was carried out under Alberta Research Permit Number 25‐188 and Saskatchewan Permit Number 25AR004 (Supporting Information: Appendix C, Appendix 1).

## Conflicts of Interest

The authors declare no conflicts of interest.

## Supporting Information

Additional supporting information can be found online in the Supporting Information section.

## Supporting information


**Supporting Information** The supporting file consists of a site detailed plan that outlines the experimental rotation details for a specific site—site A5. It also consists of Appendix A which describes camera setup for the sites, Appendix B, a site data collection sheet primarily focused on ensuring the function of the cameras, and Appendix C, the relevant wildlife permit which was required to be carried by the individuals managing the sites in Alberta. Appendix 1, within Appendix C, was supplied by the Albertan government and outlines the terms of the permit.

## Data Availability

The data that support the findings of this study are available from the corresponding author upon reasonable request.

## References

[1] Zhong K. , Chen X. , and Zhang J. , et al.Recent Advances in Oral Vaccines for Animals, Veterinary Sciences. (2024) 11, no. 8, 10.3390/vetsci11080353. PMC11360704 39195807

[2] Newton E. J. , Pond B. A. , and Tinline R. R. , et al.Differential Impacts of Vaccination on Wildlife Disease Spread During Epizootic and Enzootic Phases, Journal of Applied Ecology. (2019) 56, no. 3, 526–536, 10.1111/1365-2664.13339, 2-s2.0-85061266904.

[3] Rupprecht C. E. , Buchanan T. , and Cliquet F. , et al.A Global Perspective on Oral Vaccination of Wildlife Against Rabies, Journal of Wildlife Diseases. (2024) 60, no. 2, 241–284, 10.7589/JWD-D-23-00078. 38381612

[4] Barnett K. and Civitello D. , Ecological and Evolutionary Challenges for Wildlife Vaccination, Trends in Parasitology. (2020) 36, no. 12, 970–978, 10.1016/j.pt.2020.08.006. 32952060 PMC7498468

[5] Napper S. and Schatzl H. , Oral Vaccination as a Potential Strategy to Manage Chronic Wasting Disease in Wild Cervid Populations, Frontiers in Immunology. (2023) 14, 10.3389/fimmu.2023.1156451, 1156451. 37122761 PMC10140515

[6] Bartz J. C. , Benavente R. , and Caughey B. , et al.Chronic Wasting Disease: State of the Science, Pathogens. (2024) 13, no. 2, 10.3390/pathogens13020138. PMC10892334 38392876

[7] Napper S. and Schatzl H. , Vaccines for Prion Diseases: A Realistic Goal?, Cell and Tissue Research. (2023) 392, no. 1, 367–393, 10.1007/s00441-023-03749-7. 36764940 PMC9918406

[8] Moazami-Goudarzi K. , Andreoletti O. , Vilotte J. , and Berigue V. , Review on PRNP Genetics and Suscuptibility to Chronic Wasting Disease of Cervidae, Veterinary Research. (2021) 52, no. 1, 10.1186/s13567-021-00993-z. PMC8499490 34620247

[9] Schatzl H. , Abdelaziz D. , and Dalton C. , et al.Developing Vaccines for Chronic Wasting Disease, 2024, Taylor and Francis, 1–47.

[10] Government of Canada , Chronic Wastering Disease of Deer and Elk, 2025, [Accessed 5 November 2025] https://inspection.canada.ca/en/animal-health/terrestrial-animals/diseases/reportable/cwd.

[11] Government of Alberta , Current and Historical Alberta Weather Station Data Viewer, 2025, [Accessed 4 November 2025] https://acis.alberta.ca/weather-data-viewer.jsp.

[12] Stats Canada , Map 2, 2025, [Accessed 4 November 2025] https://www150.statcan.gc.ca/n1/daily-quotidien/250627/mc-b002-eng.htm.

[13] Government of Canada , Government of Canada Provides Update on 2025 Wildfires as Support Continues, 2025, [Accessed 4 November 2025] https://www.canada.ca/en/public-safety-canada/news/2025/10/government-of-canada-provides-update-on-2025-wildfires-as-support-continues.html.

[14] Bajrovic I. , Schafer S. , Romanovicz D. , and Croyle M. , Novel Technology for Storage and Distribution of Live Vaccines and Other Biological Medicines at Ambient Temperature, Science Advances. (2020) 6, no. 10, 10.1126/sciadv.aau4819, eaau4819. 32181330 PMC7056310

[15] Jurata , Technology, 2025, [Accessed 4 November 2025] https://www.juratatf.com/technology.

[16] Barraso P. , Lopex-Olvera J. , Kiluba T. K.w , and Gortazar C. , Overcoming the Limitations of Wildlife Disease Monitoring, Research Directions: One Health. (2024) 2, 10.1017/one.2023.16, e3.

[17] Singh S. , Sharma P. , Pal N. , Sarma D. K. , Tiwari R. , and Kumar M. , Holistic One Health Surveillance Framework: Synergizing, ACS Infectious Diseases. (2024) 10, no. 3, 808–826, 10.1021/acsinfecdis.3c00625. 38415654

[18] Bradley H. S. , Tomlinson S. , Craig M. D. , Cross A. T. , and Bateman P. W. , Mitigation Translocation as a Management Tool, Conservation Biology. (2022) 36, no. 1, 10.1111/cobi.13667, e13667. 33210780

[19] Fenichel E. , Horan R. , and Hickling G. , Management of Infectious Wildlife Diseases: Bridging Conventional and Bioeconomic Approaches, Ecological Applications. (2010) 20, no. 4, 903–914, 10.1890/09-0446.1, 2-s2.0-77953172848. 20597279

[20] Gortazar C. , Diez-Delgado I. , Barasona J. A. , Vicente J. , De La Fuente J. , and Boadella M. , The Wild Side of Disease Control at the Wildlife-Livestock-Human Interface: A Review, Frontiers in Veterinary Science. (2015) 1, 10.3389/fvets.2014.00027, 2-s2.0-84929004398. PMC4668863 26664926

[21] Manjerovic M. , Green M. , Mateus-Pinilla N. , and Novakofski J. , The Importance of Localized Culling in Stabilizing Chronic Wasting Disease Prevalence in White-Tailed Deer Populations, Preventative Veterinary Medicine. (2014) 113, no. 1, 139–145, 10.1016/j.prevetmed.2013.09.011, 2-s2.0-84888206046. 24128754

[22] Roux S. and Reist A. , Killed For Good: Hunters, Biologists, and the Ethical Paradoxes of Wildlife Management in North America, Journal of Contemporary Ethnography. (2024) 53, no. 5, 663–688, 10.1177/08912416241265875.

[23] Cunningham A. , Daszak P. , and Wood J. , One Health, Emerging Infectious Diseases and Wildlife: Two Decades of Progress?, Philosophical Transactions of the Royal Society B: Biological Sciences. (2017) 372, no. 1725, 10.1098/rstb.2016.0167, 2-s2.0-85020459691, 20160167. PMC5468692 28584175

[24] Dobson A. , Pimm S. L. , and Hannah L. , et al.Ecology and Economics for Pandemic Prevention, Science. (2020) 369, 379–381, 10.1126/science.abc3189. 32703868

[25] Holmes E. , COVID-19—Lessons for Zoonotic Disease, Science. (2022) 375, 1114–1115. 35271309 10.1126/science.abn2222

[26] Koomans M. , Behravesh C. B. , and Cunningham A. A. , et al.The Panzootic Spread of Highly Pathogenic Avian Influenza H5N1 Sublineage 2.3.4.4b: A Critical Appraisal of One Health Preparedness and Prevention, The Lancet Infectious Diseases. (2024) 24, e774–e781. 39134084 10.1016/S1473-3099(24)00438-9 PMC12096394

[27] Benn J. S. , Nunez C. M. , and Blue-McLendon A. , et al.Lethal Toxin Neutralizing Antibody Response Induced Following Oral Vaccination With a Microencapsulated *Bacillus Anthracis* Sterne Strain 34F2 Vaccine Proof-of-Concept Study in White-Tailed Deer (*Odocoileus virginianus*), Journal of Zoo and Wildlife Medicine. (2024) 55, no. 1, 212–218, 10.1638/2023-0065. 38453505

[28] Tibbs-Cortes B. W. , Rahic-Seggerman F. M. , Schmitz-Esser S. , Boggiatto P. M. , Olsen S. , and Putz E. J. , Fecal and Vaginal Microbiota of Vaccinated and Non-Vaccinated Pregnant elk Challenged With *Brucella abortus* , Frontiers in Veterinary Science. (2024) 11, 10.3389/fvets.2024.1334858, 1334858. 38352039 PMC10861794

[29] VerCauteren K. , Feuka A. , and Lavelle M. , et al.Oral Delivery of Bovine Tuberculosis Vaccine to Free-Ranging White-Tailed Deer, Frontiers in Veterinary Science. (2025) 12, 10.3389/fvets.2025.1548627, 1548627. 39995549 PMC11847888

[30] Jiménez-Cabello L. , Utrilla-Trigo S. , Lorenzo G. , Ortego J. , and Calvo-Pinilla E. , Epizootic Hemorrhagic Disease Virus: Current Knowledge and Emerging Perspecitves, Microorganisms. (2023) 11, no. 5, 10.3390/microorganisms11051339, 1339. 37317313 PMC10224379

[31] Niroula N. , Ghodasara P. , and Marreros N. , et al.Orally Administered Live BCG and Heat-Inactivated Mycobacterium bovis Protect Bison Against Experimental Bovine Tuberculosis, Scientific Reports. (2025) 15, 10.1038/s41598-025-88176-0, 3764. 39885300 PMC11782570

[32] Sharma G. , Milne V. , and Wallis D. , Vaccination of Pronghorn Antelope (*Antilocapra americana peninsularis*) With an Inactivated Bluetongue Virus Vaccine Elicits Virus-Neutralized Antibody Response and Possible Protection Under Field Consitions, Journal of Zoo and Wildlife Medicine. (2026) 57, no. 1, 20–30, 10.1638/2024-0011. 41926250

[33] Masse A. and Cote S. , Spatiotemporal Variations in Resources Affect Activity and Movement Patterns of White-Tailed Deer (*Odocoileus virginianus*) at High Density, Canadian Journal of Zoology. (2013) 91, no. 4, 252–263, 10.1139/cjz-2012-0297, 2-s2.0-84876146572.

[34] Northrup J. , Anderson C. , Gerber B. , and Wittemyer G. , Behavioral and Demographic Responses of Mule Deer to Energy Development on Winter Range, Widlife Monographs. (2021) 208, no. 1, 1–37, 10.1002/wmon.1060.

[35] Tøien O. , Blake J. , Edgar D. M. , Grahn D. A. , Heller H. C. , and Barnes B. M. , Hibernation in Black Bears: Independence of Metabolic Suppression from Body Temperature, Science. (2011) 331, no. 6019, 906–909, 10.1126/science.1199435, 2-s2.0-79951824325. 21330544

[36] Berry S. , Shipley L. , Long R. , and Loggers C. , Differences in Dietary Niche and Foraging Behavior of Sympatric Mule and White-Tailed Deer, Ecosphere. (2019) 10, no. 7, 10.1002/ecs2.2815, 2-s2.0-85069891742, e02815.

[37] Center for Disease Control , Chronic Wasting Disease, 2025, [Accessed 5 November 2025] https://www.cdc.gov/chronic-wasting/data-research/index.html.

[38] Fehler-Gardiner C. , Rudd R. , Donovan D. , Slate D. , Kempf L. , and Badcock J. , Comparing ONRAB and RABORAL V-RG Oral Rabies Vaccine Field Performance in Raccoons and Striped Skunks, Journal of Wildlife Diseases. (2012) 48, no. 1, 157–167, 10.7589/0090-3558-48.1.157, 2-s2.0-84856027485. 22247384

