# Supplementary material for: Evaluating Potential Deployment Strategies for Oral Delivery of Vaccines for Cervids
Source: Transbound Emerg Dis. 2026 May 28;2026:5900650. doi: 10.1155/tbed/5900650 (PMC13216712; doi:10.1155/tbed/5900650)
Supplement: Supplementary file 1 — Supporting Information The supporting file consists of a site detailed plan that outlines the experimental rotation details for a specific site—site A5. It also consists of Appendix A which describes camera setup for the sites, Appendix B, a site data collection sheet primarily focused on ensuring the function of the cameras, and Appendix C, the relevant wildlife permit which was required to be carried by the individuals managing the sites in Alberta. Appendix 1, within Appendix C, was supplied by the Albertan government and outlines the terms of the permit. [file TBED-2026-5900650-s001.pdf]

Saskatoon, SK • Canada • S7K 3J7 Tel 306.934.8784

Site Specific Information

Site Designation: A5
Site Description: Liquid Delivery and Mineral Licks
Site Representative: Alberta Conservation Association
Site Location: Alberta
Site Timeframe: May 26 - Aug 29, 2025

1. Experimental Overview

This experiment aims to evaluate wildlife response to different bait types with the long-term goal of determining an optimal deployment method for a CWD Vaccine.

2. Site Setup Instructions

2.1 Materials Provided By OHMT

1 × Feed bucket
1 × Tie strap
2 × 7.0 lb containers of Boba spheres
1 × Mineral lick with installation cord

2.2 Initial Setup

Detailed site set-up details can be found in Appendix A.

a) Ensure you are carrying the required Research and Collection Permit (Appendix C) for all field activities
b) Read and review class protocol #011 for Ground-Based Wildlife Surveys (https://open.alberta.ca/publications/class-activity-ground-based-wildlife-surveys)
c) Select a suitable location with moderate wildlife traffic and minimal human disturbance.
d) Install the first camera 4-5 meters from the feeding station, at a height of 0.75-1 meter.
e) Ensure the camera has an unobstructed view of the feeding area.
f) Clear a 2-meter radius around the feeding station of excessive debris or vegetation.
g) Install the second camera 1-2 meters from the feeding station, at an angle, providing a view into the feed bucket.

2.3 Bucket Installation

A bucket and bucket tie strap are included in your materials kit.

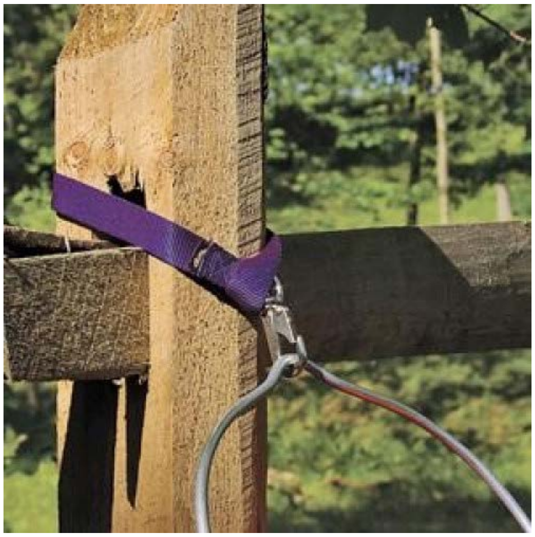

a) Select a sturdy tree/post with a diameter > 10 cm.

b) Wrap the tie strap around the tree/post at a height of 0.5 meters from the ground.
c) Clip the tie strap to the bucket handle, ensuring it's secure. Optimally the bucket will sit 5-10 cm elevated from the ground with the tie strap taut to prevent spillage.
d) Test the stability by gently pushing the bucket to ensure minimal movement.

<u>2.4 Mineral Lick Installation</u>

a) When called for in the schedule, hang the mineral lick 0.5-0.75 meters above the bucket.
b) Use the provided cord to secure the mineral lick to the same tree/post in view of Camera 2.

3. Experimental Treatments

<u>3.1 Treatment Descriptions</u>

a) Control — Site fully set-up with bucket in place, but no bait in the feeder
b) Boba — Pour half of one container of Boba solution (3.5 lbs/ ~1.6 litres) into the feed bucket, ensuring a good distribution of liquid and boba.
c) Mineral Lick — Install the supplied mineral lick, hanging it as described in section 2.4. Remove when not called for in the schedule.
d) Mineral Lick + Boba — Combination of treatments (b) and (c), with both elements installed simultaneously.

<u>3.2. Experimental Schedule</u>

Note: While exact start and end dates for each period can be adjusted based on your availability, please maintain the two-week duration as much as possible for each treatment. Notify the research team of any schedule changes.

PERIOD	DATES	TREATMENT
**1**	May 26 – June 9	Control
**2**	June 9 - June 23	Boba
**3**	June 23 – July 7	Boba+ Mineral Lick
**4**	July 7 – July 21	Mineral Lick
**5**	July 21 – Aug 4	Control
**6**	Aug 4 – Aug 18	Boba
**7**	Aug 18 – Aug 29	Boba+ Mineral Lick

4. Data Collection

A data collection sheet is provided in Appendix B; however, this sheet is primarily required during initial set-up or following a camera change. Following visits only require recording the following as a quick message to azimmerling@onehealthmedtech.com or info@onehealthmedtech.com:

a) Date and time of visit
b) Bait consumption estimate (%)
c) Any extreme evidence of wildlife visitation (bear activity, significant digs etc.)
d) Any maintenance performed
e) Any unusual observations or concerns

5. Safety and Compliance Guidelines

<u>5.1 Volunteer Liability Disclaimer:</u>

Participation in this research study is entirely voluntary. One Health Medical Technologies assumes no responsibility or liability for any injuries, accidents, or incidents that may occur during site setup, maintenance,

or monitoring activities. By participating in this research, volunteers acknowledge they are conducting these activities at their own risk and accept full personal responsibility for their safety and well-being while performing study-related tasks. Volunteers should exercise appropriate caution, follow all safety guidelines provided, and use proper judgment when conducting fieldwork. This research does not include any form of insurance coverage or compensation for volunteers in case of injury or accident.

5.2 Safety Guidelines

a) Follow all relevant organizational safety processes and protocols (i.e. check-ins, carrying safety equipment etc.)
b) Maintain appropriate distance if wildlife is present upon arrival. Use caution, as baits may attract other species such as bears.
c) Do not attempt to handle or approach wildlife.
d) Report any concerning wildlife behavior to your supervisor immediately, and then One Health Medical Technologies as soon as possible.

5.2 Regulatory Compliance

This research is conducted under Wildlife Research Permit 029055984. Ensure a signed copy of this permit is carried during all field activities related to this project.

6. Contact Information

Primary Contact: Amanda Zimmerling
Work: 1-306-934-8784
Cell: 1-780-884-0729
Email: azimmerling@onehealthmedtech.com

Appendix A

CWD Vaccine Deployment Preliminary Testing- Site Set-Up Protocol

1) Locate a likely feed site location by identifying areas of suspected deer activity (game trails, bedding sites etc.) With the goal of each site sampling an independent population, place sites >5 km apart whenever possible.
2) Determine an optimal location for feed placement – each site will be specified as elevated or ground placement, so determine an area to hang/place the feed vessel as required.
3) Identify a suitable attachment point for Camera 1 in the vicinity of the target area (e.g., tree, fence post) that supports:
   a) a detection zone ~4-5 m from the camera (~4-5 m from the feed placement),
   b) a Field of View (FOV) ~ 5 m wide and 10 m long (unobstructed by objects, shrubs or trees),
   c) aims the camera perpendicular to the expected movement path
   d) faces the camera north or south
4) Identify a suitable attachment point for Camera 2 in the vicinity of the target area (e.g., tree, fence post) that supports:
   a) a detection zone ~1 m from the feed placement, and ~0.5-1 m above the feed placement
   b) aims the camera slightly downward to primarily view into the feed vessel, or directly view the feed site
   c) faces the camera north or south
5) Trim vegetation as needed to reduce likely false triggers.

Note: It may be necessary to bring a man-made attachment point (e.g., stake).

6) Before setting up the camera, record the Camera Make and Camera Model, Camera Serial Number, and optionally the Camera ID, SD Card ID, key ID (for python or cable lock), attachment and the equipment that will be used to secure the camera.
7) Ensure the SD card is inserted, the batteries are fresh and turn the camera on.
8) Check (and record) the camera settings
   a. Trigger Mode(s) - Motion
   b. Image/Video Mode – Images
   c. Trigger Sensitivity- High
   d. # of Photos Per Trigger – 1
   e. Motion Image Interval – 5 sec
   f. Date and Time – ensure correctness
   g. Record the Deployment Start Date
9) Attach and secure the camera to the tree/post (e.g., security box or bracket, cable lock and lock box, as needed). Security / lock boxes are recommended to avoid theft.
   a. Cameras should be slightly angled downward and facing north when possible, south when not, to reduce sun glare.
10) Perform a walktest to confirm that both cameras activate for activity at the feed site.
   a. Activate the walktest mode.
   b. Attach the camera at the desired camera height, angle, and direction.
   c. Walk in front of the cameras to the feed site and wave your hand in front of the cameras to determine if both cameras are activating.
11) Arm the cameras or wait for the camera to arm itself (~2 minutes of inactivity).
12) Record the deployment metadata (specifically, Sample Station Name, Camera Location Name, Deployment Name, Deployment Crew, and Deployment Start Date on a Test Image Sheet (Appendix B, page 7)

13) Walk ~5 m in front of the camera. Face the Test Image Sheet towards the camera and slowly walk towards the camera. If the Test Image Sheet is laminated, tilt it slightly downward to avoid sun glare on the shiny surface. Allow the camera to take a series of images. Relevant deployment metadata should be documented in this way each time the feed site is serviced.

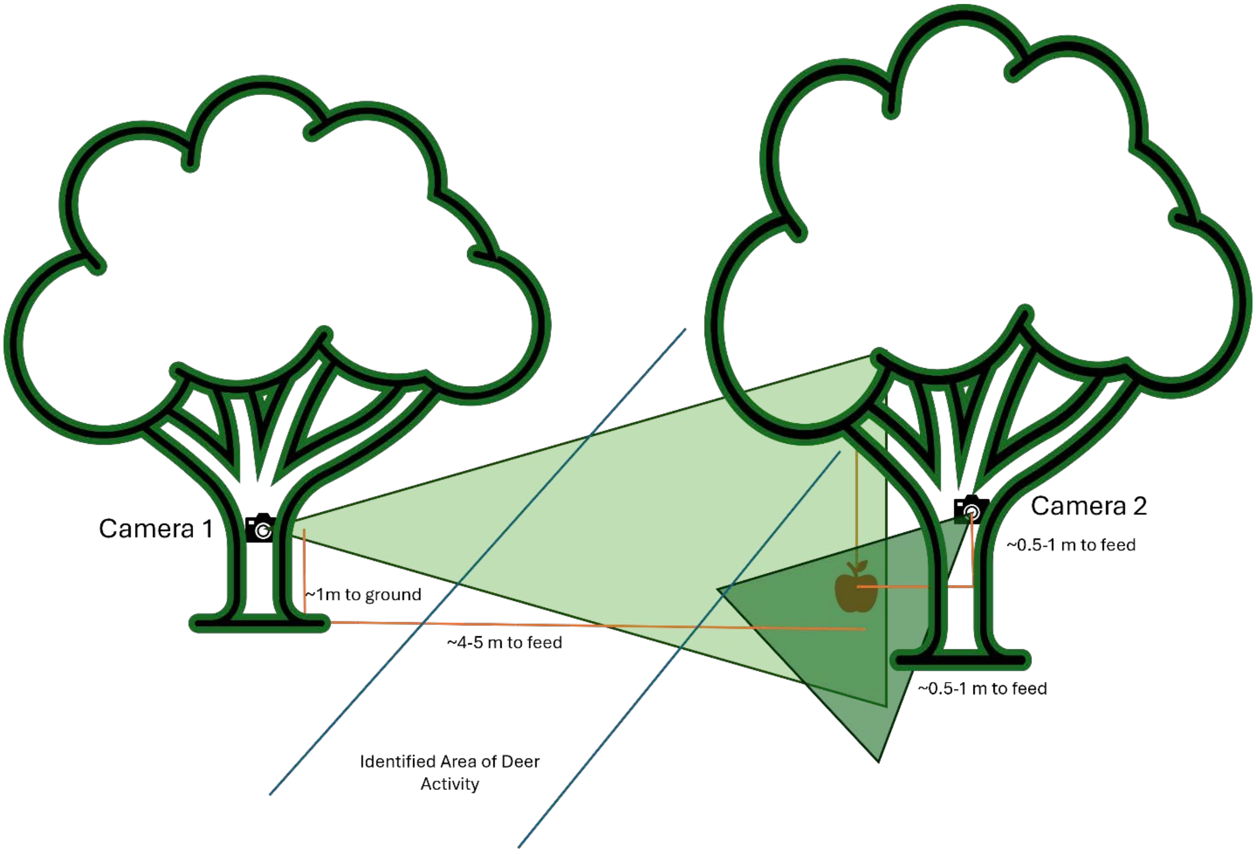

Appendix B
Data Collection Sheet: CWD Vaccine Preliminary Research Summer 2025

Date:_______________________________

Site ID:_____________________________

Site Name:__________________________

Personnel: ___________________________________

Camera Information
Camera 1
Camera ID/Serial Number:____________ Camera Make:__________________

Camera Settings

a. Trigger Mode(s) – Motion ☐
b. Image/Video Mode – Images ☐
c. Trigger Sensitivity- High ☐
d. # of Photos Per Trigger – 1 ☐
e. Motion Image Interval – 5 sec ☐
f. Date and Time – ensure correctness ☐
g. Record the Deployment Start Date _________________________

Camera 2
Camera ID/Serial Number:_____________ Camera Make:_______________________

Camera Settings

a. Trigger Mode(s) – Motion ☐
b. Image/Video Mode – Images ☐
c. Trigger Sensitivity- High ☐
d. # of Photos Per Trigger – 1 ☐
e. Motion Image Interval – 5 sec ☐
f. Date and Time – ensure correctness ☐
g. Record the Deployment Start Date _________________________

Walk Test Performed: Yes/No
Notes:

DATE:

PERSONNEL:

STATION ID:

LOCATION:

Appendix C
Alberta Wildlife Research and Collection Permit

Alberta

#25-188

General Permit - GP

RESEARCH PERMIT
FEE $ NIL

Region: North Saskatchewan/Red Deer

PERMITTEE: Amanda Zimmerling, One Health Medical Technologies

ADDRESS: 1 Cory Place, East Cory Industrial Park, Saskatoon, SK S7K 3J7

IS AUTHORIZED TO: bait deer at camera traps to evaluate delivery systems for chronic wasting disease vaccines.

DATE OF ISSUE: March 14, 2025

DATE OF EXPIRY: August 31, 2025

Amanda Zimmerling Digitally signed by Amanda Zimmerling DN: cn=Amanda Zimmerling, o, ou, email=aszimmerling@gmail.com, c=CA Date: 2025.04.03 10:53:55 -06'00'

Signature of Permittee

S. Stevens

Scott Stevens, Senior Wildlife Biologist
Alberta Environment and Parks
Red Deer Office
North Saskatchewan/Red Deer Region

IN ACCORDANCE WITH: The approved research plan (application # 29055984) and Class Protocol(s) #011

Class Protocols are reviewed by the Alberta Wildlife Animal Care Committee and approved by the Director of Fish and Wildlife Policy.Class Protocols are available at http://aep.alberta.ca/fish-wildlife/wildlife-research-collection/default.aspx.

Conditions:

1. The Permit is subject to all conditions listed in the attached Appendix 1.
2. The permit holder is responsible for all individuals conducting activities under this permit.
3. Landholder permission required prior to any baiting/camera trap activities.
4. All baits and equipment must be removed at the end of the study period.
5. No hunting over bait-stations.

Alberta

#25-188

Licence - CN

COLLECTION LICENCE
FEE $ NIL

Region: North Saskatchewan/Red Deer

NAME: Amanda Zimmerling, One Health Medical Technologies

ADDRESS: 1 Cory Place, East Cory Industrial Park, Saskatoon, SK S7K 3J7

Is authorized, subject to the conditions of this licence, and in accordance with the approved research plan # 29055984, to hunt* or collect, the following wildlife species: mule deer, white-tailed deer

This Licence authorizes the use of the following equipment and methods: Trail cameras, various feed materials.

This Licence is valid (location): Lower Peace, Upper Athabasca, Lower Athabasca, North Saskatchewan/Red Deer, South Saskatchewan

EFFECTIVE DATE: April 14, 2025

DATE OF EXPIRY: August 31, 2025

Hunting and/or collection is to be conducted by: Amanda Zimmerling, Numerous citizen scientists, hunters, private landowners, outfitters and conservation organizations

Date of issue: March 14, 2025

Signature of Licencee (not valid until signed by the Licencee)

Licence must be carried while hunting or collecting.

S. Stevens

Scott Stevens, Senior Wildlife Biologist
Alberta Environment and Parks
Red Deer Office
North Saskatchewan/Red Deer Region

**The meaning of the word "hunt" is inclusive of activities within the definition of "hunt" in the Wildlife Act except that the activity authorized by this licence is limited to what is expressly stated in it and, to be lawful, that activity must occur in accordance with the approved research plan and licence conditions.*

Appendix 1: Research Permit General Permit Conditions
Addendum to Research Permit #25-188

1. The permit is valid only for research and collection activities in the specific area and for the dates identified on the permit.
2. All research must be conducted according to the approved Research Plan (the approved application) along with any special conditions outlined on the permit and/or licence; all captured animals must be handled in a humane manner and according any additional approvals or instructions provided by the Alberta Wildlife Animal Care Committee.
3. Animals captured using immobilization drugs must follow the Chemical Immobilization of Wildlife: Drug Volume Calculation Tables: https://www.alberta.ca/system/files/custom_downloaded_images/ep-chemical-immobilization-drug-calculation.pdf
4. For all projects, a report of activities from the past year is required before permits will be renewed. If the project has finished and the permit will not be renewed, the report is due within 30 days of the expiry of the permit. Reports are to be uploaded via the Online Permitting and Clearance system (OPaC) https://www.opac.alberta.ca/, and must include:

   a) A Progress Report with a general summary of project activities
   b) All wildlife observations made during the project, uploaded via OPaC using either:

   i) a FWMIS Load Form (for the appropriate data type), or
   ii) where USFWS bands are used in the project, a BANDIT digital export. Note: Banding data locations are to be provided as Latitude/Longitude in Degrees-Minutes-Seconds.

   FWMIS.xls digital files can be accessed at the following web site:
   https://www.alberta.ca/wildlife-loadforms.aspx

   c) Any accidental mortalities, oversampling exceeding 10%, and incidental captures.
   d) If radio telemetry is a component of the research, the report must include the following information:

   i) frequencies used, date, general location, species, transmitter type, manufacturer, and expected transmitter life.
   ii) last known whereabouts of transmitters still deployed.
5. Notwithstanding the authorization that this permit confers, while conducting wildlife research activities, the permit holder is responsible for the following:
   a) For activities in any Provincial Park, Provincial Recreation Area, Wildland Provincial Park, Willmore Wilderness Park, Ecological Reserve, Heritage Rangeland, Natural Area, or Wilderness Area, additional approvals for access may be required; please contact your local Alberta Environment and Protected Areas, Parks Division authority or visit https://albertaparks.ca/albertaparksca/science-research/
   b) The issuance of this Permit does not exempt the permit holder from any other Canadian Laws that might otherwise apply, including, but not limited to, requirements under the federal Migratory Birds Convention Act or Species at Risk Act.
   c) This Permit does not exempt the Permit Holder from the need to obtain permission to access private or leased land.
   d) The Permit Holder is responsible for ensuring that public safety is not endangered by activities associated with the project.
   e) The Permit Holder may be held accountable for damages to resources or property arising directly or indirectly from the project.
